# Housing conditions affect rat responses to two types of ambiguity in a reward–reward discrimination cognitive bias task

**DOI:** 10.1016/j.bbr.2014.07.048

**Published:** 2014-11-01

**Authors:** Richard M.A. Parker, Elizabeth S. Paul, Oliver H.P. Burman, William J. Browne, Michael Mendl

**Affiliations:** Centre for Behavioural Biology, School of Veterinary Science, University of Bristol, Langford House, Langford BS40 5DU, UK

**Keywords:** Cognitive bias, Decision-making, Affect, Emotion, Rat, Pessimism

## Abstract

Decision-making under ambiguity in cognitive bias tasks is a promising new indicator of affective valence in animals. Rat studies support the hypothesis that animals in a negative affective state evaluate ambiguous cues negatively. Prior automated operant go/go judgement bias tasks have involved training rats that an auditory cue of one frequency predicts a Reward and a cue of a different frequency predicts a Punisher (RP task), and then measuring whether ambiguous cues of intermediate frequency are judged as predicting reward (‘optimism’) or punishment (‘pessimism’). We investigated whether an automated Reward–Reward (RR) task yielded similar results to, and was faster to train than, RP tasks. We also introduced a new ambiguity test (simultaneous presentation of the two training cues) alongside the standard single ambiguous cue test. Half of the rats experienced an unpredictable housing treatment (UHT) designed to induce a negative state. Control rats were relatively ‘pessimistic’, whilst UHT rats were quicker, but no less accurate, in their responses in the RR test, and showed less anxiety-like behaviour in independent tests. A possible reason for these findings is that rats adapted to and were stimulated by UHT, whilst control rats in a predictable environment were more sensitive to novelty and change. Responses in the new ambiguity test correlated positively with those in single ambiguous cue tests, and may provide a measure of attention bias. The RR task was quicker to train than previous automated RP tasks. Together, they could be used to disentangle how reward and punishment processes underpin affect-induced cognitive biases.

## Highlights

• We investigated how an unpredictable housing treatment (UHT) influenced measures of rat affect.
• Control rats showed more anxiety-like behaviour in open-field and elevated plus maze tests than UHT rats.
• Controls also made more ‘pessimistic’ decisions in an automated cognitive bias task.
• Our go/go reward–reward task was learnt faster than previous automated go/go tasks.
• We developed a new ambiguity test that may probe biases in attentional processes.

## 1 Introduction

Accurate measures of animal affect (emotion) are required in neuroscience, psychopharmacology, pain research, and animal welfare science in order to provide models of affective disorders with translational validity, knowledge about the affective component of pain, and better indicators of animal welfare. Whilst direct measures of conscious emotion are not available, behavioural and physiological components of affective states can be assessed by, for example, open field, elevated plus maze [1], [2], [3], [4] and forced swim tests [3], [5], vocalizations [1], facial expressions, and stress physiology readouts [3]. Such measures form the bedrock of animal emotion research but may have limitations including lack of cross-species generality, absence of theoretical frameworks for predicting how affective states should influence test readouts, and measurement of affective arousal but not affective valence (positivity vs. negativity) [6], [7]. A new approach, drawing on robust findings from human psychology that affective states can influence cognitive function and decision-making, may usefully address these issues. For example, people reporting negative emotional states attend more to negative stimuli (attention bias), more readily retrieve negative memories (memory bias), and are more likely to judge ambiguous stimuli negatively than happier people (a pessimistic judgement bias) – affective valence appears to be reliably linked to these ‘cognitive biases’ [3], [8], [9], [10].

Harding et al. [11] were the first to develop a method for studying affect-induced cognitive biases in non-human animals. Rats were trained to lever press in response to a tone (2 kHz or 4 kHz) to receive a food reward, and to refrain from responding when they heard a different tone (4 kHz or 2 kHz) in order to avoid a burst of white noise. Once this go/no-go conditional discrimination task had been learnt, subjects were occasionally exposed to tones intermediate in frequency between the training tones to examine whether they responded to these ambiguous cues as if they predicted the food reward (‘optimistic’ response) or the white noise (‘pessimistic’ response). Rats housed in unpredictable housing conditions that induce a mild negative depression-like state [12], [13] were less likely to judge ambiguous tones as predicting a rewarding outcome than those housed in control conditions – they showed a pessimistic-like bias in line with findings that people in negative states judge ambiguous stimuli negatively [3], [8], [9].

Harding et al.’s generic approach has been extended to a variety of other species including rhesus [14] and capuchin monkeys [15], dogs [16], [17], sheep [18], starlings [19], honeybees [20], and back-translation to humans [21], [22], [23]. In many, though not all (e.g. [17], [24], [25]), of these studies affect manipulations result in judgement biases as predicted. Affect-induced judgement biases thus show cross-species generality, measure affective valence, and can be couched in theoretical frameworks that provide a priori predictions for how affective state should influence biases [7], [26], thus making them promising new indicators of animal affect.

The most commonly studied species to date is the laboratory rat, with 10 out of 12 published studies finding predicted changes in judgement bias. A variety of automated and non-automated discrimination tasks have been used. Naturalistic operant responses (e.g. approach/avoidance; digging) have been trained in non-automated go/no-go tasks that yield Reward and Punishment (RP) or Reward and No-reward (RN) outcomes [27], [28], [29], and in less rapidly trained go/go tasks that yield two Rewarding outcomes (RR) of differing value [30], [31], [32]. Traditional ‘arbitrary’ operant responses (e.g. lever-pressing) have been used in automated go/no-go tasks [11] and go/go tasks [33], [34], [35], [36], [37] employing Rewarding and Punishing (RP) outcomes.

Unbalanced go/no-go tasks where one response (e.g. ‘go’) is always linked to the same outcome (e.g. reward) can be difficult to interpret if treatment manipulations cause non-affect related changes in general activity, or if affective valence is asymmetrically linked with activity (e.g. positive states inherently enhance vigour [38]). Furthermore, ‘no-go’ responses may sometimes simply reflect extinction of responses to ambiguous cues [39]. Therefore, the most desirable task for ease of interpretation, low experimenter time demands, and hence widespread uptake is a rapidly trained and automated go/go task. However, training to criterion on automated go/go tasks that use RP outcomes is time consuming (e.g. >26 days [33]; 40–60 days [34]; 37–52 days [36]), with training of active punishment avoidance usually taking longer than training of active reward acquisition (e.g. 13–17 vs. c.6 days [33]; 25–26 vs. 14–17 days [36]).

We therefore investigated the utility of an automated go/go task using two rewarding outcomes of differing value to avoid lengthy training of active punishment avoidance responses. Such an RR task, in combination with existing RP and RN tasks, would also facilitate investigation of judgement biases in the context of different combinations of rewards and punishers, allowing the role of underlying reward acquisition and punishment avoidance systems to be disentangled. In the present study, we employed an automated go/go active choice lever-press task using two reward outcomes – one vs. two 45 mg food pellets. We previously showed that rats would preferentially press a lever associated with a 2-pellet reward than one associated with a single pellet reward, even when they had to perform 13 times as many presses for the bigger reward (the maximum ratio tested) [40]. Rats were trained to press one lever in response to a tone predicting the larger outcome and a different lever in response to a tone predicting the smaller and less rewarding outcome. They then experienced either an unpredictable housing treatment (UHT) designed to induce a negative state, or control (predictable) housing. We hypothesised that UHT rats would show a ‘pessimistic’ cognitive bias compared to controls.

To measure judgement biases, we presented a wide range of ambiguous tones to gain a detailed picture of any treatment-related changes in the shape of the resulting generalisation curve between the two training tones. We also employed a different ambiguity test altogether, namely presentation of the two trained tones at the same time. In contrast to the standard single ambiguous tone tests which likely engage mechanisms that compare the presented tone to a tone-outcome distribution retrieved from memory, we reasoned that simultaneous presentation of both training tones would engage attentional mechanisms such that decisions might reflect biases in attention to one or other tone. Thus, the new ambiguity test might provide a useful measure of attention bias, and we compared responses across the two types of ambiguity test. We cross-referenced any treatment-related changes in the subjects’ affective and motivational state by employing tests of feeding motivation [41], [42], anhedonia (sucrose consumption tests [43]), and anxiety-like states [44], [45], [46], [47].

## 2 Material and methods

### 2.1 Subjects

The experimental subjects were 16 male Lister hooded rats (*Rattus norvegicus*; Harlan UK Ltd., Bicester, UK); they were approximately 6 months old at the start of magazine training, and were experimentally naïve. The rats were housed in stable pairs in cages measuring 56 cm (L) × 34 cm (W) × 19 cm (H), on a 12-h light cycle (lights off at 9 am). The cages contained sawdust bedding, shredded paper for nesting, a shelter and a chew block, and were cleaned on the same morning each week. All subjects had ad libitum access to food (Eurodent Diet 22%) and water. Six male Lister hooded rats, previously subjects in unrelated studies, provided the social stimuli in the unpredictable housing treatment; they were 16 months old at the start of the treatment. All the rats were checked daily for health throughout the experiment, which was conducted under UK Home Office licence 30/2249.

### 2.2 Apparatus

Two operant chambers, of identical design, were used, in two different rooms (not those in which the rats were housed). The operant chambers measured 52 cm (L) × 30 cm (W) × 35 cm (H); three of the walls and the floor were metal (the latter was covered with sawdust bedding), the long rear wall was Perspex, and wire mesh covered the ceiling. A food hopper was located centrally on the long metal wall (3.5 cm above the floor), with a retractable lever on either side (4 cm away from the side of the trough, 8 cm above the floor). The chamber was illuminated with a 1.12 W white light bulb, and a water bottle hung at the rear. Loudspeakers were placed centrally, at ceiling level above the food hopper, facing down into the chamber. Their volume was adjusted so the tonal intensity was 70 dB at the approximate location of the rat's ears when he was sitting between the levers, in front of the hopper. The house lights, levers, pellet dispensers, speakers and tone generators were manufactured by Coulbourn Instruments (Allentown, PA, USA), and operated by their Graphic State (v3.02) software. The hopper delivered Bioserv (Frenchtown, NJ, USA) Dustless Precision Pellets (45 mg).

The elevated plus maze (EPM; Coulbourn Instruments) consisted of four arms at right angles to each other, each 50 cm (L) × 10 cm (W), connected by a central hub allowing the rat to move between them. The two ‘closed’ arms were on opposite sides of the hub, and had vertical walls, 30 cm high, either side of the runway (but not at their terminal end). The other two arms had a small raised edge, and no walls. The maze was made of black, opaque Perspex, and was illuminated by two 60 W red lights. It was raised 55 cm from the floor. The open field was a square, Perspex arena, with white, opaque walls and floor, and a transparent roof. The walls were 30 cm (H) × 65 cm (L). A transparent plastic food container filled with sand constituted the novel object. It was square, with a sealed lid, and measured 6 cm (H) × 11 cm (L). The arena was illuminated by a 60 W red light.

### 2.3 Treatment

Eight of the rats underwent unpredictable changes in their husbandry regime designed to be mildly stressful (unpredictable housing treatment: UHT). This procedure was devised by Harding et al. [11] and based on a ‘chronic mild stress’ procedure designed to induce an ‘anhedonic’ state in rodents [12]. The treatment lasted for 28 days, and consisted of five different husbandry events which could occur at any time during the dark phase of the rats’ light cycle, excluding a period 2 h before, or after, any testing or training. No more than two events occurred on any given day, and they did not overlap. The events, with the maximum duration (h) and maximum frequency (per week) in parentheses, were as follows: bedding dampened by 100 ml water (12 h, 1/wk); light cycle reversed (2 h, 3/wk); cage tilted by 30° (7 h, 2/wk); rat placed in cage containing odour of unfamiliar conspecific (4 h, 3/wk); rat placed in home cage of unfamiliar conspecific with that conspecific present (2 h, 3/wk; this event was terminated if damaging aggression was seen to occur). The remaining eight experimental subjects constituted the control group; their husbandry regime underwent no such changes.

### 2.4 Procedure

Rats were trained on the judgement bias task and then tested with ambiguous probe tones, and on other tests (see below), during a baseline Phase 1 prior to experiencing treatment manipulations. During and at the end of the treatment period (Phase 2), they received another set of ambiguous probe tone tests together with other tests. Precise timings are provided in the following sections.

#### 2.4.1 Judgement bias task – magazine training

All rats received six sessions of magazine training. Each session started with the presentation of one lever which, if pressed, resulted in the immediate delivery of one food pellet. If ten lever presses were made, the lever was retracted, and the other lever was presented and reinforced on the same schedule. This alternating pattern continued until the rat had made 60 lever presses, or 60 min had elapsed, whichever came first. In addition, regardless of any lever-pressing, a parallel autoshaping schedule was in operation in which the active lever automatically retracted for 1 s prior to the delivery of one food pellet. This procedure occurred every minute during the first three sessions and every 3 min during the final three sessions.

#### 2.4.2 Judgement bias task – discrimination training

Following magazine training, the rats received training sessions, each of which lasted 40 min. The subjects were presented with a series of trials consisting of a 2-s presentation of a reference auditory tone (either 2 kHz or 4 kHz), followed by the presentation of both (free-choice) or one (forced-choice) lever(s). As soon as the rat pressed a lever, the lever(s) were retracted, and food was delivered, as appropriate. By default, the trials were free-choice (both levers available), and each auditory tone (2 kHz or 4 kHz) had an equal probability of presentation. If the rat pressed the correct lever (i.e. the one designated by the experimenter as corresponding to the tone presented), either 1 or 2 pellets of food were delivered, depending on the identity of the stimulus. If the rat pressed the incorrect lever, no food was delivered, and the following trial was forced-choice (only one lever available): i.e. the same auditory tone was played again, and only the correct lever was presented. The inter-trial interval (ITI) was 30 s.

The rats received one training session per day, with an occasional day off, until they reached criterion. Criterion was defined as three consecutive sessions in which performance in free-choice trials was significantly greater than chance, as judged by a binomial test, for each trial type [e.g. 11]. Once a rat had reached this criterion, it received two further sessions over the next two days, before the amount of training it received was reduced to a lower rate (once or twice per week, depending on performance) until the last rat had reached criterion. This allowed all subjects to start the treatment at the same time thus avoiding the possible confound of prolonged solitary housing (e.g. whilst cage-mates underwent UHT manipulations) for rats slower to reach criterion.

Once all the rats had reached criterion, the design of the operant task was changed so that it more closely resembled later probe sessions: the forced-choice trials were dropped, the probability of reinforcement following a correct response was reduced to 0.75 [e.g. 48], and the ITI was decreased to 15 s. The order of tone presentation was pseudo-randomised to guard against long sequences of the same stimulus. The number of sessions the rats received with this modified task again depended on performance, but all received two sessions on consecutive days immediately prior to ambiguous probe-testing (see below) before the treatment was applied, and three sessions on consecutive days immediately prior to probe-testing during the treatment (these are hereafter referred to as ‘refresher’ training sessions).

#### 2.4.3 Judgement bias task – ambiguous probe-testing

The rats received two types of ambiguous probe-testing: presentation of single tones of a frequency different to the reference tones with which they were trained (single-frequency probes), and simultaneous presentation of the two reference tones (dual-frequency probes). The rats received six single-frequency probe testing sessions: three on consecutive days before the treatment began (Phase 1 – baseline data) and three on consecutive days during the treatment (Phase 2 – tests starting 18 days into the UHT). They received two dual-frequency probe testing sessions: one on the day following each block of three single-frequency test sessions.

The single-frequency probe testing sessions terminated after 156 trials, or 60 min had passed, whichever came first. In 50% of the trials, the reference tones were presented (2 kHz and 4 kHz, with equal probability across the session); otherwise, one of 13 probe tones was presented (again, with equal probability across the session). The probe tones ran from 1.6 kHz to 4.4 kHz in 200 Hz increments, meaning there were nine probe tones of a frequency intermediate to the two reference tones, and two at either far end of the range. All correct responses to the reference tones were reinforced with food, except for a subset of each reference tone which occurred with the same probability as trials for each probe tone, which were not. The dual-frequency probe-testing session ended after 30 min had passed, or 64 trials had been completed, whichever came first. In 50% of trials, a single reference tone was presented (either 2 kHz or 4 kHz, with equal probability across the session). In the other 50% of trials, both reference tones were presented simultaneously. Correct responses to single reference tone trials were usually reinforced with food, except for a random subset (25%), which were not.

All tones were presented for 2 s, the ITI was 15 s, and trial order was pseudorandomised. All trials were free-choice; once a response had been made, both levers were retracted, the ITI commenced, and food was immediately delivered, if appropriate. Responding in probe trials was never reinforced with food. The operant chamber was cleaned with 70% ethanol solution, dried, and the sawdust replaced, prior to each training and testing session. The reference tones, lever position, quantity of food reinforcement, room in which the operant training and testing took place, and prospective treatment group assignment were all counterbalanced.

#### 2.4.4 Sucrose preference test

Individual sucrose preference tests [e.g. 43] were conducted once before the start (following judgement bias probe-testing), and at the end (Day 28), of the treatment. Rats were tested in cages fitted with two pre-weighed drink bottles, one containing water, the other 1% sucrose solution. The test lasted 3 h; the bottles’ position was swapped halfway through, and at the end they were weighed again. In addition, sucrose preference tests were also conducted in rats’ home-cages; their water bottles were removed at the start of the test and replaced with two drink bottles, one containing water, the other 1% sucrose solution. The procedure thereafter was the same as for the individual test, except that the bottles were weighed and reversed after 4 h, with the test lasting a total of 8 h. These tests were conducted once before the start of the treatment, on two occasions during it (days 7 and 14), and once on the day after it ended.

#### 2.4.5 Feeding motivation test: time taken to eat 50 pellets of food

The time the rats took to eat 50 pellets of food was tested twice: once before the start (following probe-testing) of the treatment, and once on the day following the end of the treatment. Rats were tested individually, in their home cage. A bowl containing 50 food pellets (as used in their operant training and testing) was placed in the cage, and the time from when they first took a pellet into their mouth until all were eaten was recorded. This test was designed to measure feeding motivation.

#### 2.4.6 Feeding motivation test: lever pressing progressive ratio test

A progressive ratio test with food reinforcement was conducted once, towards the end (Day 24) of the treatment. Rats were tested individually in the operant chambers, although one of the subjects in the control group was excluded from this test due to ill-health on the day. The session started with the lever associated with the two pellet reward being presented. The first lever press resulted in the immediate delivery of two pellets, and thereafter presses were reinforced on a progressive ratio of five. If no reinforcement took place for 5 min, or 60 min had elapsed, the session terminated. Such progressive schedules have been employed elsewhere as indicators of feeding motivation [41], [42].

#### 2.4.7 Elevated plus maze (EPM), open field and novel object tests

All rats were tested in an EPM on the final day of the treatment. Each rat was placed in the centre, facing the same closed arm, and then filmed for 5 min. The rat's location in the arena (with respect to the central hub and each arm) was recorded, and the total number of crossings across area boundaries, the percentage of test session time spent in the open arms, and the latency to first enter an open arm, were then calculated. In addition, the percentage of test session time spent performing grooming, rearing, and head dipping from the open arms, and the number of discrete bouts of each, was also recorded.

An open field test followed by a novel object test was administered on the day before the EPM. Each rat was placed into the empty arena in the same corner, facing the centre, and then filmed for 15 min. The first 5 min constituted the open field test. The rat then spent a further 5 min in the arena before a novel object was placed in the centre, and the rat's behaviour recorded for a final 5 min (novel object test). For the open field test, the rat's location in the arena (divided into nine squares of equal area: one central and eight peripheral) was recorded. From these observations, the total number of areas entered, and the percentage of test session time spent in the central area of the arena, was calculated. In addition, the percentage of test session time spent grooming, and rearing, were also recorded, together with the number of discrete bouts of each [e.g. 46]. For the novel object test, the arena was divided into a central square (43 cm long, containing the novel object with an 11 cm boundary of floor space surrounding it) and the remaining peripheral area, and the rat's location during the test session, with respect to these two areas, was recorded. From these observations, the percentage of test session time spent in the peripheral area (i.e. away from the novel object) was calculated.

The video footage from the EPM, open field and novel object test was analysed using Observer (v5.0) software (Noldus, Wageningen, The Netherlands) by a volunteer blind to treatment assignment. In all tests the rat was judged to have entered an area when all four paws were in it. All the apparatus was sprayed with 70% ethanol solution, and wiped dry, before each rat's session. Rats were tested individually, in a counterbalanced order.

#### 2.4.8 Bodyweights

The rats’ bodyweights were measured at five time points: before, three times during (on days 7, 14 and 21), and after the treatment (the day following its termination).

### 2.5 Data analysis

The values of the single-frequency probe (and reference) stimuli were log-transformed prior to analysis so that the intervals between them increased in magnitude as the tonal frequencies (Hz) to which they corresponded decreased in value, resulting in a closer fit to the likely psychophysical character of the auditory stimuli used [49]. This scale was standardised around the quantity of the associated food reinforcer, so that the values of ‘1’ and ‘2’ corresponded to the tonal frequencies associated with one and two pellets of food, respectively. Since the reference stimuli were not at the terminal ends of the scale presented to the subjects, the presented tone was included in statistical models up to a cubic power when used as a predictor.

The lever choice and latency data from the judgement bias task refresher training and probe sessions were analysed using repeated measures multilevel (a.k.a. mixed) models in MLwiN v.2.30 [50]. The models were either 2-level (in the case of the dual-frequency data, with trial nested within subject) or 3-level (in the case of the single-frequency probe and refresher training data, with session as an additional level intermediate to trial and subject). When probe value was added as a predictor in the analyses of the single-frequency data, by default (i.e. model convergence allowing) its coefficient (up to the quadratic term) was allowed to randomly vary at the session and subject-level (allowing for subject- and session-level variation in the shape of the generalisation curve); otherwise, the intercept was allowed to vary randomly at all levels in all models. Other predictors were added to structure the data as per hypotheses, and/or to investigate possible confounds.

Any trial in which a lever press was not recorded was excluded from the analysis (omissions occurred at extremely low rates: 0.16% for single-frequency probe trials, and 0% for dual-frequency probes), and all linear continuous predictor variables were centred around their grand mean. The analyses of the single-frequency probe data included only those trials which could not be reinforced by food (i.e. including a subset of reference trials). Lever choice was analysed as a binary response in a model with a logit link, estimated using second-order penalised quasi-likelihood (PQL2) procedures [e.g. 51]. The latency to press a lever was analysed as a continuous response variable; to better meet model assumptions this was negative reciprocal root (−1/√*y*) transformed. If the residual plots from these models suggested any persistent non-normality, a sensitivity analysis was conducted comparing models fitted both with and without any potential outliers (since this revealed no difference in the substantive findings, estimates from the analysis of the full dataset are reported in Section 3). All other analyses reported met the assumptions of the statistical procedures used, following transformations as appropriate. Wald tests and (where appropriate) likelihood ratio tests (LRT) were used to test the significance of predictors. Variables entered into models are listed in Section 3 and graphs of results show predictions derived from significant models; full estimates from these multilevel models are reported as supplementary data (see electronic supplementary material (ESM)).

All other analyses were conducted in SPSS 14.0 [52]. For the repeated measures GLMs, treatment was a between-subject factor, and measurement occasion (or phase) a within-subject factor. When measurement occasion had more than 2 levels, we followed the advice of Quinn and Keough [53] and rejected the null hypothesis if either the adjusted univariate output, or the multivariate output, reported significance at the 0.05 level.

## 3 Results

### 3.1 Judgement bias task – discrimination training

In the final session of magazine training, all rats pressed the levers 60 times, and each ate all the pellets dispensed (bar two who left one each). All subjects reached the initial criterion of three successive sessions in which the number of correct responses in free-choice trials was significantly greater than chance for both reference stimuli in a binomial test. The mean number of training sessions to reach criterion was 26.6 (SEM: 1.4), and there was no significant difference between prospective treatment groups in the number of pre-criterion (*t*_14_ = 0.04, *p* = 0.966, *d* = 0.02), nor post-criterion (*t*_14_ = −0.95, *p* = 0.357, *d* = −0.48), training sessions, nor in the probability of responding correctly in the refresher training sessions prior to the first phase of probe-testing (all trials: *Wald* = 0.13, 1*df*, *p* = 0.716 (see Model 1 in the electronic supplementary material (ESM) for full estimates from the model); ‘2-pellet’ tone trials only: *Wald* = 0.01, 1*df*, *p* = 0.947 (Model 2); ‘1-pellet’ tone trials only: *Wald* = 0.09, 1*df*, *p* = 0.764 (Model 3)). Furthermore, inclusion of the number of training sessions as a predictor in the analyses modelling responses to probe-testing, described below, did not change any of the substantive findings, nor did the inclusion of time-related predictors, such as session number and trial number.

### 3.2 Judgement bias task – responses to single-frequency probes

**Fig. 1:**
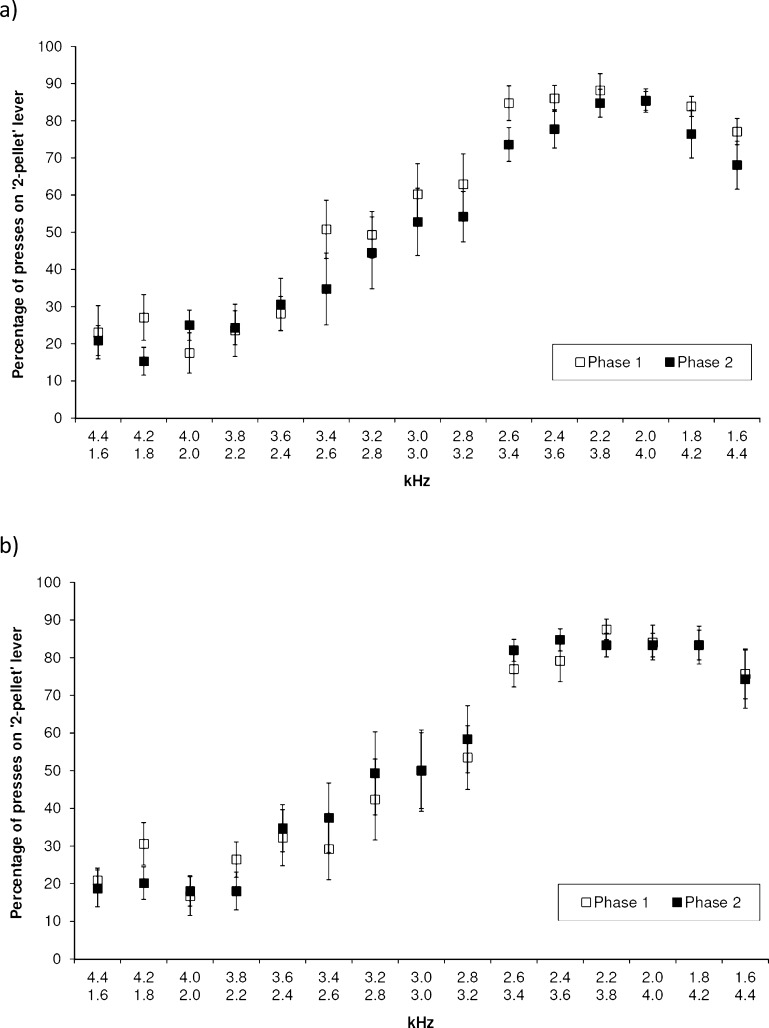
The mean percentage of lever presses made on the lever associated with 2 pellets (as opposed to 1 pellet) of food by measurement phase (Phase 1 = baseline) for the (a) Control and (b) UHT group. Upper *x*-axis: 2 kHz = 2 pellet tone; lower *x*-axis: 4 kHz = 2 pellet tone. The means and SEM are derived from data summarised at the subject-level; the data for the ‘reference tones’ (i.e. 2 kHz and 4 kHz) pertain to non-reinforced trials only.

Fig. 1 plots the mean percentage of presses on the 2-pellet lever for each treatment group in each measurement phase. The probability of pressing the 2-pellet (rather than 1-pellet) lever across measurement phase was found to significantly differ between the two treatment groups in a model including probe value, tone-outcome contingency group, its interaction with probe value, treatment group and phase (*Wald* = 3.98, 1*df*, *p* = 0.046; Model 4 in ESM). The model indicated that the probability of the control group pressing the 2-pellet lever decreased from Phase 1 to Phase 2, whilst there was little change in the probability of the UHT group doing so. When interactions between treatment, measurement phase and probe value were added, the terms were not significant (*Wald* = 4.91, 3*df*, *p* = 0.178; Model 5 in ESM), indicating that the shape of the generalisation curve did not differ by treatment, across phase.

**Fig. 2:**
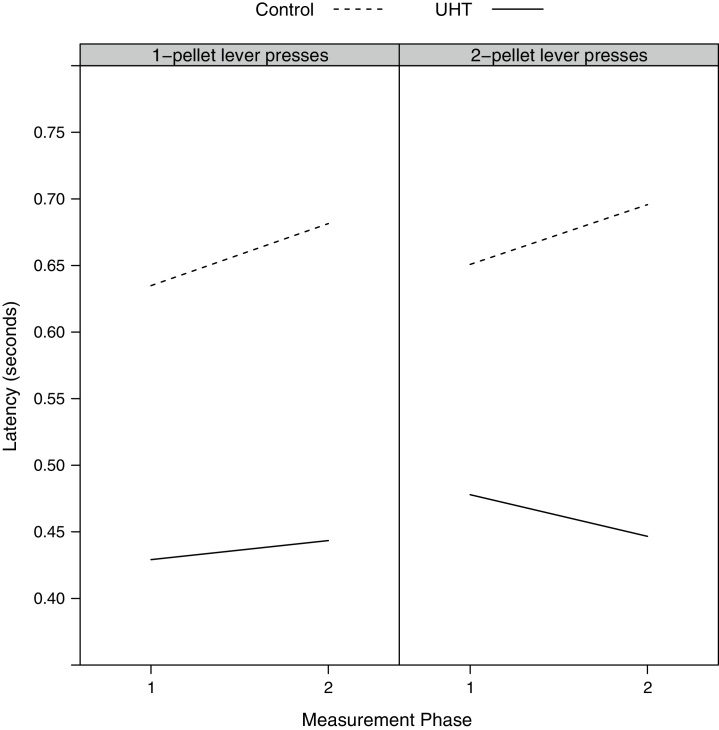
Mean predicted latency (back-transformed) to press the 1-pellet lever, and the 2-pellet lever, by treatment, across measurement phase (Phase 1 = baseline), derived from the single-frequency probe testing model discussed in the text.

**Fig. 3:**
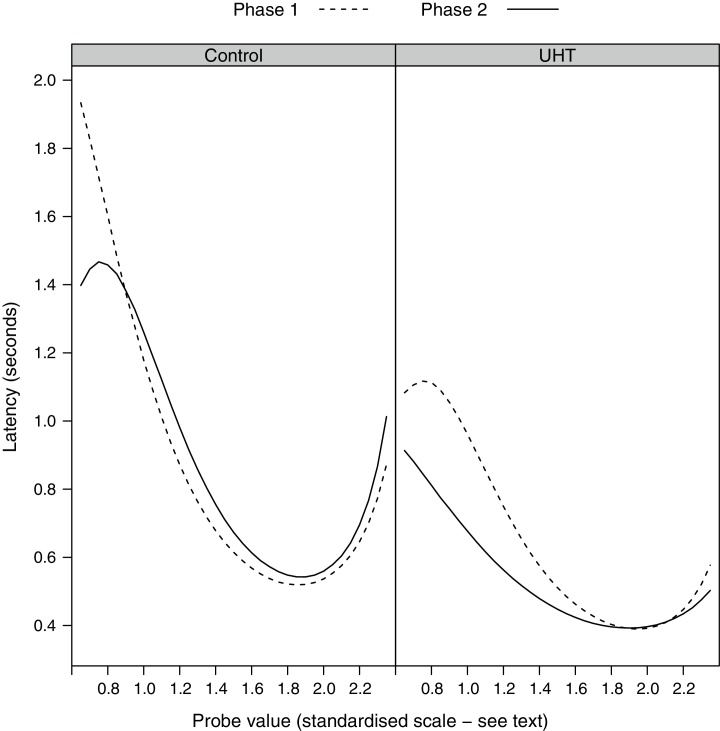
Mean predicted latency (back-transformed) to press the 2-pellet lever, by treatment, across measurement phase (Phase 1 = baseline) and probe value, derived from the single-frequency probe testing model discussed in the text.

When the latency to press a lever was analysed as the response variable (in a model containing terms for probe value, contingency group, whether the lever pressed was associated with 1 pellet or 2 pellets, their interactions, treatment group and phase), there was no significant difference, by treatment, in the latency to respond across phase (LRT = 1.91, 1*df*, *p* = 0.167; Model 6 in ESM). However, the addition of further interactions between phase, treatment and whether the lever pressed was associated with 1 pellet or 2 pellets did significantly improve the fit of the model (LRT = 8.44, 3*df*, *p* = 0.038; Model 7 in ESM); this indicated that the UHT group were quicker to press the 2-pellet lever in Phase 2 compared to Phase 1, whilst the control group were slower to do so, whereas both groups showed a similar small increase in latency to press the 1-pellet lever from Phase 1 to Phase 2 (Fig. 2). When analysing only those presses on the 2-pellet lever, the latency to respond at different probe values significantly differed across phase depending on which treatment group the rats were in (LRT = 8.35, 3*df*, *p* = 0.039; Model 8 in ESM); inspection of the plotted model predictions indicated that the UHT rats became quicker to press the lever in response to probe values around the 1-pellet reference tone in Phase 2, with little difference in treatment-related response latencies to other stimuli across phase (Fig. 3). The equivalent interaction term in a model investigating only those presses on the 1-pellet lever was not significant (LRT = 4.22, 3df, *p* = 0.238; Model 9 in ESM).

### 3.3 Judgement bias task – responses to dual frequency probes

The probability of pressing the 2-pellet lever in response to the dual-frequency probe stimuli showed a non-significant tendency to decrease from Phase 1 to Phase 2 in the control group, but little change across phase in the UHT group (*Wald* = 2.81, 1*df*, *p* = 0.094 (Model 10 in ESM); also containing terms for contingency, treatment and phase). UHT rats also tended to decrease their lever-press latency more than the control group across phase (LRT = 3.81, 1*df*, *p* = 0.051 (Model 11 in ESM); after controlling for contingency, the identity of the lever pressed, their interaction, treatment and phase). The addition of further interactions between treatment, phase and the lever pressed (1 pellet or 2 pellet) did not significantly predict latency (LRT = 4.62, 3*df*, *p* = 0.202; Model 12 in ESM). The grand means for each rat of the probability of pressing the 2-pellet lever during single-frequency and dual frequency probe tests were positively correlated in both Phase 1 (*r* = 0.561, *N* = 16, *p* = 0.024) and Phase 2 (*r* = 0.873, *N* = 16, *p* < 0.001), indicating that individuals responded similarly under both testing conditions.

### 3.4 Refresher training sessions – responses to reference stimuli

**Fig. 4:**
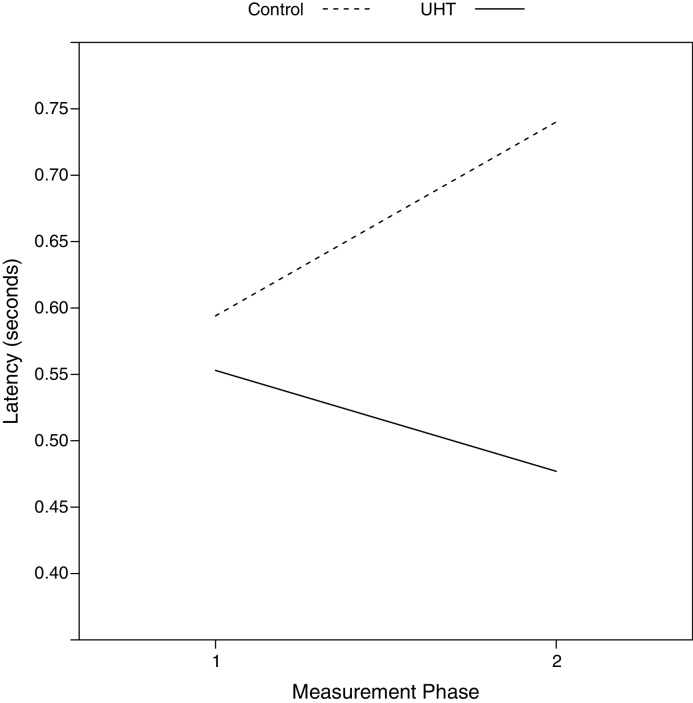
Mean predicted latency (back-transformed) to press a lever in response to the reference stimuli in the refresher training sessions, by treatment, across measurement phase (Phase 1 = baseline), derived from the model discussed in the text.

For the refresher training sessions occurring just prior to the Phase 1 and Phase 2 test sessions, whether the lever pressed was correct was analysed as a response variable in a model which controlled for the reference stimulus (‘2-pellet’ or ‘1-pellet’) presented, contingency, their interaction, treatment and phase. Adding an interaction between treatment and phase was not significant (*Wald* = 1.18, 1*df*, *p* = 0.277; Model 13 in ESM), nor was the interaction between phase, treatment, and reference stimuli (*Wald* = 0.66, 1*df*, *p* = 0.417; Model 14 in ESM). When modelling latency as the response variable in a model (containing terms for contingency, the stimulus presented (‘2-pellet’ or ‘1-pellet’), whether a response was correct or not, their interactions, treatment and phase), the interaction between phase and treatment was significant, indicating that the UHT group were faster to respond, and the Control group slower, in Phase 2 compared to Phase 1 (LRT = 22.12, 1*df*, *p* < 0.001 (Model 15 in ESM); Fig. 4).

### 3.5 Feeding motivation tests

Rats had a shorter latency to eat 50 pellets of food during Phase 2 than Phase 1 (*F*_1,14_ = 6.29, *p* = 0.025, $\eta_{p}^{2}=0.31$), but there were no significant treatment or interaction effects (treatment: *F*_1,14_ = 1.46, *p* = 0.248, $\eta_{p}^{2}=0.09$; treatment * phase: *F*_1,14_ = 0.04, *p* = 0.854, $\eta_{p}^{2}=0.01$). With regard to the lever pressing progressive ratio test, all sessions terminated before 60 min had elapsed. The total number of times the lever was pressed during the progressive ratio test did not differ between treatments (*t*_13_ = 0.289, *p* = 0.778, *d* = 0.15).

### 3.6 Sucrose preference test

In the individual test, a repeated-measures GLM did not detect any significant effects of treatment (*F*_1,14_ = 0.05, *p* = 0.833, $\eta_{p}^{2}=0.01$), phase (*F*_1,14_ = 1.05, *p* = 0.323, $\eta_{p}^{2}=0.07$) and their interaction (*F*_1,14_ = 0.03, *p* = 0.869, $\eta_{p}^{2}=0.01$) on the amount of sucrose solution consumed as a percentage of total fluid (i.e. water and sucrose solution) intake. In the home-cage test, treatment did not affect sucrose consumption (*F*_1,6_ = 2.717, *p* = 0.150, $\eta_{p}^{2}=0.31$), and the GLM adjusted univariate output revealed no treatment * phase interaction (*F*_1.05,6.33_ = 0.048, *p* = 0.846, $\eta_{p}^{2}=0.01$) but did reveal an effect of phase (*F*_1.05,6.33_ = 5.767, *p* = 0.050, $\eta_{p}^{2}=0.49$). Inspection of plotted data indicated that the consumption of sucrose solution increased from a low value in the first test session to maintain an approximate plateau across the next three test sessions.

### 3.7 Bodyweight

**Fig. 5:**
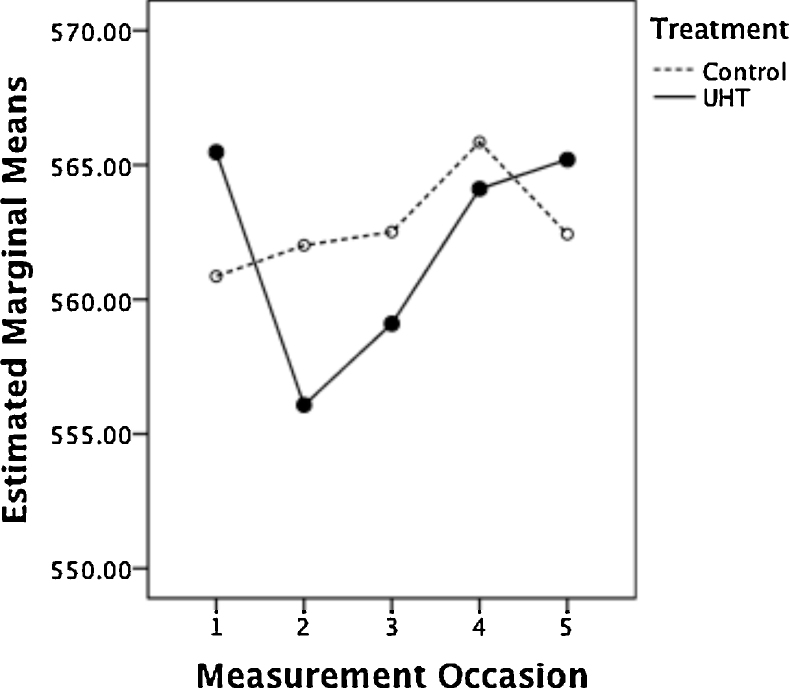
The estimated marginal means of bodyweight (in g), by treatment, across time (where 1 = pre-treatment, 2–4 = during treatment, 5 = day following end of treatment), derived from the repeated-measures GLM discussed in the text.

Inspection of the multivariate and adjusted univariate output from a repeated-measures GLM showed that measurement occasion was significant at the 0.05 level in both, whilst the interaction between measurement occasion and treatment was significant only in the multivariate output (measurement occasion: *F*_4,11_ = 10.31, *p* = 0.001, $\eta_{p}^{2}=0.79$; measurement occasion * treatment: *F*_4,11_ = 3.95, *p* = 0.032, $\eta_{p}^{2}=0.59$; all multivariate output); treatment, as a main effect, was not significant (*F*_1,14_ = 0.01, *p* = 0.967, $\eta_{p}^{2}=0.01$). Fig. 5 indicates that bodyweight of the UHT group decreased at the start of the treatment, in contrast to bodyweight of the control group, which increased slightly during this period, as reflected in the finding that the within-subjects interaction contrast was highly significant between the pre-treatment measurement and that taken after seven days of the treatment (*F*_1,14_ = 10.73, *p* = 0.006, $\eta_{p}^{2}=0.44$), whilst the other interaction contrasts were not significant (*p* > 0.05).

### 3.8 Elevated plus maze (EPM), open field and novel object tests

**Fig. 6:**
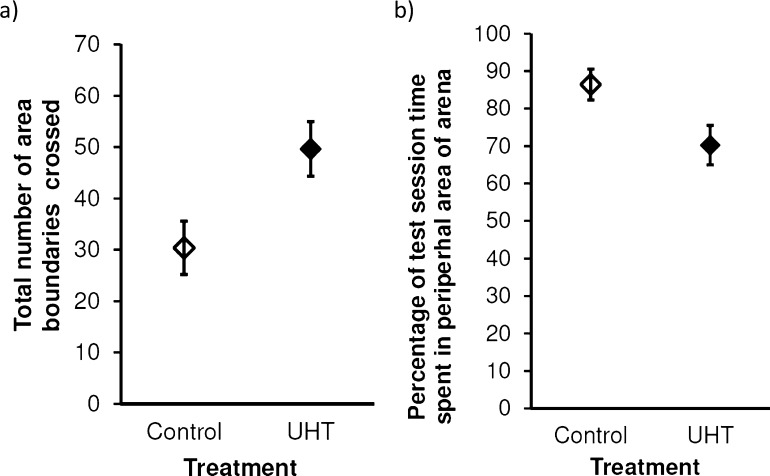
The mean (a) number of area boundaries crossed in the open field test, and (b) percentage of time spent in the peripheral area of the arena following addition of a novel object into the central area, by treatment (error bars = 1 SEM).

Rats in the UHT group tended to spend more time in the open arms of the EPM than those in the control group (*t*_14_ = −2.09, *p* = 0.056, *d* = −1.04), whilst the latency to first enter an open arm (*t*_14_ = 1.12, *p* = 0.282, *d* = 0.56), the total number of head dips (*t*_14_ = −1.71, *p* = 0.109, *d* = −0.86), the total number of area boundary crossings (*t*_14_ = −1.22, *p* = 0.244, *d* = −0.61), the time spent rearing (*t*_14_ = −1.57, *p* = 0.139, *d* = −0.79), and the time spent grooming (*U* = 39.0, *z* = 0.75, *p* = 0.505, *r* = 0.19) did not differ. In the open field test, UHT rats crossed significantly more area boundaries than control rats (*t*_14_ = −2.59, *p* = 0.022, *d* = −1.29; Fig. 6a), although there was no difference in the time they spent in the central square (*U* = 25.0, *z* = −0.74, *p* = 0.505, *r* = −0.19), the time they spent grooming (*t*_14_ = 1.22, *p* = 0.243, *d* = 0.61), nor the time they spent rearing (*t*_14_ = −0.42, *p* = 0.682, *d* = −0.21). Following the introduction of the novel object to the central area of the arena, control rats did spend significantly more time than UHT rats in the peripheral area (*t*_14_ = 2.23, *p* = 0.043, *d* = 1.12; Fig. 6b), although there was no significant difference in the (log-transformed) latency to contact the novel object (*t*_14_ = −0.09, *p* = 0.931, *d* = −0.04) nor in the percentage of test session time spent in contact with it (*t*_14_ = −1.58, *p* = 0.136, *d* = −0.79).

## 4 Discussion

### 4.1 Evidence for affect-induced judgement bias

We found that control group rats were significantly less likely to perform ‘optimistic’ 2-pellet lever presses in response to single-frequency probe stimuli in phase 2 than in phase 1, compared to UHT rats who showed little change in the probability of 2-pellet lever presses across phases. A non-significant trend indicated a similar pattern for the dual-frequency probe stimuli. These results do not support our main hypotheses, and run contrary to other studies which have found that animals undergoing a treatment designed to induce a more negative/less positive affective state are likely to respond to ambiguous stimuli in a manner in keeping with a more ‘pessimistic’/less ‘optimistic’ judgement of their outcome. They either suggest that the cognitive bias test used here was unable to detect changes in affect that were induced by the treatments as predicted, or that the treatments had a counter-intuitive effect on the rats’ affective state. To differentiate between these two possibilities, it is instructive to review the other measurements we took to try to better establish the nature of any change in affect.

### 4.2 Effects of treatment on measures of food motivation, anhedonia, anxiety and body weight

There were no significant effects of treatment on the sucrose preference test (designed to detect anhedonia), or in the two tests devised to measure changes in food motivation, indicating that treatment-induced differences in reward (food) valuation were unlikely to be responsible for the observed results. The UHT group crossed significantly more area boundaries in an open field arena, spent significantly more time in close proximity to a novel object, and had a near-significant tendency to spend more time on the open arms of an elevated plus maze (EPM), compared to controls. These tests are widely used for rats – ‘anxious’ animals are assumed to have a greater tendency to avoid exposure (e.g. stay close to walls (thigmotaxis); seek cover), and to stay away from novel elements [44], [45], [46], [47] – and thus suggest, counter-intuitively, that UHT rats were in a less anxious and more exploratory state than control animals, perhaps because they had become accustomed to a changing and variable environment where new and unexpected events are commonplace.

Compared to controls, the UHT group lost a significant amount of bodyweight during the first seven days of the treatment, but then their weight steadily increased to reach its pre-treatment value. The relationship of putative stress with changes in bodyweight is somewhat equivocal [54], [55], [56], but stress is typically associated with a lower rate of weight gain, or indeed weight loss [57], [58] which can recover as animals appear to adapt to initially stressful events [59], [60].

On this basis, the data suggest that the UHT treatment may have been initially stressful, but that the level of perceived stress abated as the rats adapted, or habituated, to it. Whilst the ‘unpredictable’ nature of the UHT treatment is designed, in part, to ensure against any habituation or adaptation, there are, nevertheless, predictable elements (e.g. treatments only occur in the dark phase; a finite number of events are repeated). In addition, the treatment regime used in this study was milder than many employed elsewhere [12], [43], [55].

### 4.3 Effects of treatment on response speed in the judgement bias test

Across the two phases, rats in the UHT group became quicker, and controls slower, to lever-press. This was significant in the refresher training sessions (i.e. when responding to reference stimuli) prior to each ambiguous probe test period, and with regard to 2-pellet lever presses in the single-frequency probe sessions. There was a near-significant trend in the same direction in the dual-frequency probe sessions. Increases in response speed often come at the expense of accuracy [61], [62], but there was no evidence that UHT rats showed a decrease in accuracy during refresher training. If UHT rats were in a more negative state, this finding is contrary to human studies which indicate that depression and anxiety are generally associated with slower responses in cognitive-behavioural tasks [63], [64], [65], [66], and decreased accuracy [67], although there are various exceptions to these findings [e.g. 64].

### 4.4 Synthesis

The overall behavioural profiles of the two treatment groups ran contrary to our hypothesis. Compared to the control group who showed more ‘pessimistic’ responding across time, the UHT rats did not change their responses to ambiguous probes, decreased their response latency without any obvious loss of accuracy, lost weight but then regained it during the UHT treatment, and had a lower tendency to seek cover/avoid novelty in the EPM, open field and novel objects tests. One possible explanation for these findings is that rats adapted to the UHT treatment over time such that the stimulation associated with the relatively mild UHT procedures resulted not in a conservation/withdrawal depression-like state [68], but in elevated general arousal, decreased neophobia and a bolder response style, reflected in faster but accurate responding in the judgement bias tests and low anxiety-like behaviour. Controls, living in a relatively unchanging environment, may have been more negatively affected by novelty and change in the tests, resulting in more ‘pessimistic’ decisions and enhanced anxiety-like behaviour relative to UHT rats. Indeed there are suggestions that highly predictable environments may be stressful and that an ‘intermediate’ level of predictability, particularly for appetitive events, is desirable [69], [70], [71]. Consideration of the potential monotony of some standard housing conditions may thus be warranted in order to improve welfare.

A conceptual framework for the findings is provided by the Yerkes–Dodson law which describes an inverted-U shaped relationship between arousal or stress on the *x*-axis and (cognitive) performance on the *y*-axis [61]. Although the law is purely descriptive, and agnostic with regard to underlying mechanisms, it is nevertheless a pervasive empirical generalisation and it is recognised that ‘intermediate’ levels of stress may enhance functioning [61], [72]. UHT rats may have been closer to the peak of the inverted-U than control rats for reasons discussed above. If this is correct, a stronger environmental manipulation such as the conventional chronic mild stress paradigm [12] on which UHT was based, might be expected to shift animals further to the right on the inverted U curve and towards slower and less accurate responding and less ‘optimism’. Similar responses might also be expected in animals at an earlier stage of UHT treatment prior to any habituation or adaptation, and it is noteworthy that Harding et al. [11], who did observe less ‘optimistic’ decisions under ambiguity in UHT rats compared to controls, started judgement bias testing only 9 days after the onset of the UHT treatment, whereas testing started on day 18 of treatment in the current study. There was thus less time in Harding et al.’s study for rats to adapt or habituate to the UHT treatment, and this difference could reconcile the findings of the two studies. We should add that our findings do not mean that the UHT treatment can reasonably be conceived as ‘enriching’, especially in its conventional less mild form [12], [43], [55]; there was no evidence of a change across phase to a more ‘optimistic’ response style in UHT rats.

### 4.5 Use of the reward–reward task

Training to criterion on the go/go reward–reward (RR) task was generally faster (mean of 27 sessions) than on go/go tasks with rewarding and punishing (RP) outcomes (>26 days [33]; 40–60 days [34]; 37–52 days [36]). The RR task did detect differences in judgement of ambiguity, although these were not as predicted, and they contrasted with the findings of Harding et al. [11] who used a very similar UHT regime. Harding et al. used a go/no-go RP task involving a reward (food) and a punisher (burst of white noise). It is possible that the UHT affect manipulation, involving a variety of potentially threatening events, impacts primarily on punishment avoidance systems and, as such, increases expectation of punishment under ambiguity without decreasing anticipation of reward [7]. If so, an RR task would not be expected to detect the predicted judgement bias, but an RP task would. This might be an alternative or additional explanation to that discussed in Section 4.4 for the disparity between the present findings and those of Harding et al.

More generally, the development of RP and now RR go/go paradigms opens the way for future studies to test functional and mechanistic hypotheses relating to the roles of reward acquisition and punishment avoidance systems in generating judgement biases. For example, a pure depression-like state associated with loss/lack of reward might be predicted to induce reduced expectation of reward under ambiguity but have no effect on anticipation of punishment, whilst a pure anxiety-like state associated with increased threat of punishment might increase expectation of punishment under ambiguity without an effect on anticipation of reward (see [7], [73]). Mechanistically, it would be possible to investigate whether and how manipulation of neural systems that code the value of rewards and punishers (e.g. dopaminergic, opioidergic, serotonergic, mesocorticolimbic circuitry [38], [74], [75] affect reward-related cognitive biases (using RR and RN tasks), and punishment-related biases (using RP and PN (punishment/no-punishment) tasks).

### 4.6 Single and dual frequency ambiguous probe tests

The close positive correlations between responses to the single and dual frequency probe were striking. At a theoretical level, if simultaneous presentation of both training tones (dual-frequency probes) engages attentional mechanisms such that decisions reflect biases in attention to one or other tone, whilst presentation of a single ‘ambiguous’ tone engages different mechanisms such as retrieval of tone-outcome distributions from memory accompanied by, for example, signal-detection or drift-diffusion/race model decision processes [76], the current results indicate that both sets of processes were similarly affected by the experimental treatments. Future research should clarify the extent to which responses in dual-frequency probe tests associate with other measures of attention bias, and can be dissociated from responses in single-frequency probe tests. Lack of dissociation would indicate that both tests are tapping the same mechanism. If this is the case, then the dual-frequency probe test may be a quick and simple practical alternative to the more lengthy and complex single-frequency tests. If responses to the two types of ambiguity test do dissociate, it may be possible to use the dual-frequency test to specifically investigate attention biases which may be more closely linked to anxiety as opposed to depression-like states [3], [9].

It is also worth noting here that the shape of the generalisation curves during single-frequency probe testing did not differ across sessions, indicating that responses to probes were not systematically influenced by prior experience of these types of trial. Previous studies, especially those using go/no-go tasks, have suggested that animals may learn that probes are not reinforced and hence extinguish responses to these stimuli – this may be interpreted, incorrectly, as a ‘no-go’ response [39]. Our study used a go/go task without any time limit on responding and this ‘forced choice’ procedure required the animal to make a decision. The utility of ‘forced-choice’ procedures is well-recognised in the human psychological literature. For example, when ‘forced’ to respond, uncertain participants often perform above chance revealing decisions/knowledge that would otherwise remain ‘silent’ (e.g. [77]).

## 5 Conclusions

We found that control rats were relatively ‘pessimistic’ and slower to respond in two types of cognitive bias task, and showed higher anxiety-like behaviour in other tests, compared to UHT animals. These counter-intuitive findings may have occurred because UHT rats adapted to and were stimulated by the unpredictable housing treatment, whilst control rats living in a predictable environment became more sensitive to novelty and change. Convergence of results from different tests of affect supports this interpretation, but we cannot completely rule out the alternative possibility that the tests failed to detect changes in affect induced by UHT treatment. The automated reward–reward cognitive bias task used here was faster to train than comparable go/go reward–punishment tasks, and opens the way for studies of the role of reward acquisition and punishment avoidance processes in affect-induced cognitive biases. Use of reward–reward tasks is also in line with 3Rs recommendations to refine scientific procedures to minimise potential suffering (NC3Rs: http://www.nc3rs.org.uk/page.asp?id=7). Ambiguity tests involving simultaneous presentation of training tones correlated strongly with tests using a single ambiguous tone presentation and could be used in future as new measures of attention bias.
