# Supplementary material for: Housing conditions affect rat responses to two types of ambiguity in a reward–reward discrimination cognitive bias task
Source: Behav Brain Res. 2014 Nov 1;274:73–83. doi: 10.1016/j.bbr.2014.07.048 (PMC4199117; doi:10.1016/j.bbr.2014.07.048)
Supplement: Supplementary file 1 [file mmc1.docx]

Housing conditions affect rat responses to two types of ambiguity in a reward-reward discrimination cognitive bias task

Richard M.A. Parker, Elizabeth S. Paul, Oliver H.P. Burman, William J. Browne, Michael Mendl

*Centre for Behavioural Biology, School of Veterinary Science, University of Bristol, Langford House, Langford, BS40 5DU, UK*

**Supplementary Electronic Material**

**Estimates from multilevel models reported in the main article**

*Reference (base) categories for categorical predictors were as follows:*

- Contingency: 2kHz = 2 pellets
- Treatment: Control
- Measurement phase: Phase 1 (baseline)
- Lever pressed: 1-pellet lever
- Reference stimuli: tone associated with 1-pellet of food
- Correct?: Incorrect response

Note: the coefficient estimates (and their standard errors) reported below are not back-transformed. All continuous predictor variables (bar (standardised probe scale)^2^ and (standardised probe scale)^3^) are centred around their grand mean.

| **Response:** Correct? / **Link:** logit | | **Estimate (SE)** |
| --- | --- | --- |
| **No. of observations: n(subject)** = 16**; n(session)** = 32**; n(trial)** = 4901 | |  |
| **FIXED PART** | | **MODEL 1** |
| Β_0_ | Intercept | 1.161 (0.174) |
| Β_1_ | Contingency | -0.326 (0.202) |
| Β_2_ | Treatment | -0.071 (0.194) |
| Β_3_ | Reference stimuli | 0.854 (0.11) |
| Β_4_ | Contingency.Reference stimuli | -0.251 (0.145) |
| **RANDOM PART** | |  |
| σ^2^*_v_*_0_ | Subject-level variance | 0.124 (0.054) |
| σ^2^*_u_*_0_ | Session-level variance | 0.012 (0.018) |

| **Response:** Correct? / **Link:** logit | | **Estimate (SE)** |
| --- | --- | --- |
| **No. of observations: n(subject)** = 16**; n(session)** = 32**; n(trial)** = 2452 | |  |
| **FIXED PART** | | **MODEL 2** |
| Β_0_ | Intercept | 2.047 (0.208) |
| Β_1_ | Contingency | -0.623 (0.237) |
| Β_2_ | Treatment | -0.018 (0.236) |
| **RANDOM PART** | |  |
| σ^2^*_v_*_0_ | Subject-level variance | 0.142 (0.083) |
| σ^2^*_u_*_0_ | Session-level variance | 0.052 (0.054) |

| **Response:** Correct? / **Link:** logit | | **Estimate (SE)** |
| --- | --- | --- |
| **No. of observations: n(subject)** = 16**; n(session)** = 32**; n(trial)** = 2449 | |  |
| **FIXED PART** | | **MODEL 3** |
| Β_0_ | Intercept | 1.24 (0.282) |
| Β_1_ | Contingency | -0.343 (0.325) |
| Β_2_ | Treatment | -0.098 (0.325) |
| **RANDOM PART** | |  |
| σ^2^*_v_*_0_ | Subject-level variance | 0.353 (0.150) |
| σ^2^*_u_*_0_ | Session-level variance | 0.060 (0.046) |

| **Response:** Lever pressed / **Link:** logit | | **Estimates (SE)** | |
| --- | --- | --- | --- |
| **No. of observations: n(subject)** = 16**; n(session)** = 96**; n(trial)** = 8626 | |  |  |
| **FIXED PART** | | **MODEL 4** | **MODEL 5** |
| Β_0_ | Intercept | 0.097 (0.149) | 0.139 (0.164) |
| Β_1_ | Standardised probe scale | 6.072 (0.304) | 6.298 (0.422) |
| Β_2_ | (Standardised probe scale)^2^ | 0.975 (0.401) | 0.827 (0.522) |
| Β_3_ | (Standardised probe scale)^3^ | -8.142 (0.575) | -8.543 (0.823) |
| Β_4_ | Contingency | 0.78 (0.171) | 0.78 (0.173) |
| Β_5_ | Contingency.Standardised probe scale | -2.037 (0.414) | -2.054 (0.413) |
| Β_6_ | Contingency.(Standardised probe scale)^2^ | -2.78 (0.563) | -2.799 (0.563) |
| Β_7_ | Contingency.(Standardised probe scale)^3^ | 3.393 (0.779) | 3.434 (0.782) |
| Β_8_ | Treatment | -0.221 (0.156) | -0.358 (0.196) |
| Β_9_ | Measurement phase | -0.274 (0.108) | -0.32 (0.131) |
| Β_10_ | Treatment.Measurement phase | 0.307 (0.154) | 0.515 (0.186) |
| Β_11_ | Treatment.Standardised probe scale |  | -0.276 (0.497) |
| Β_12_ | Treatment.(Standardised probe scale)^2^ |  | 0.754 (0.608) |
| Β_13_ | Treatment.(Standardised probe scale)^3^ |  | 0.466 (0.967) |
| Β_14_ | Measurement phase.Standardised probe scale |  | -0.545 (0.404) |
| Β_15_ | Measurement phase.(Standardised probe scale)^2^ |  | 0.046 (0.425) |
| Β_16_ | Measurement phase.(Standardised probe scale)^3^ |  | 0.842 (0.918) |
| Β_17_ | Treatment.Measurement phase.Standardised probe scale |  | 0.846 (0.572) |
| Β_18_ | Treatment.Measurement phase.(Standardised probe scale)^2^ |  | -0.953 (0.607) |
| Β_19_ | Treatment.Measurement phase.(Standardised probe scale)^3^ |  | -1.187 (1.306) |
| **RANDOM PART** | |  |  |
| **Subject-level covariance matrix:** | |  |  |
| σ^2^*_v_*_0_ | | 0.081 (0.041) | 0.083 (0.042) |
| σ^2^*_v_*_01_ | | -0.14 (0.062) | -0.139 (0.061) |
| σ^2^*_v_*_1_ | | 0.289 (0.134) | 0.284 (0.132) |
| *σ^2^_v_*_02_ | | -0.063 (0.096) | -0.069 (0.097) |
| *σ^2^_v_*_12_ | | 0.403 (0.19) | 0.402 (0.189) |
| σ^2^*_v_*_2_ | | 0.706 (0.399) | 0.718 (0.396) |
| **Session-level covariance matrix:** | |  |  |
| σ^2^*_u_*_0_ | | 0.081 (0.033) | 0.079 (0.032) |
| σ^2^*_u_*_01_ | | 0.071 (0.036) | 0.067 (0.036) |
| σ^2^*_u_*_1_ | | 0.096 (0.077) | 0.077 (0.074) |
| *σ^2^_u_*_02_ | | -0.031 (0.087) | -0.006 (0.084) |
| *σ^2^_u_*_12_ | | -0.087 (0.115) | -0.068 (0.109) |
| σ^2^*_u_*_2_ | | 0.448 (0.361) | 0.345 (0.345) |

| **Response:** -1/√Latency(seconds) / **Link:** identity | | **Estimates (SE)** | |
| --- | --- | --- | --- |
| **No. of observations: n(subject)** = 16**; n(session)** = 96**; n(trial)** = 8626 | |  |  |
| **FIXED PART** | | **MODEL 6** | **MODEL 7** |
| Β_0_ | Intercept | -1.299 (0.074) | -1.293 (0.075) |
| Β_1_ | Standardised probe scale | 0.397 (0.075) | 0.394 (0.075) |
| Β_2_ | (Standardised probe scale)^2^ | 0.326 (0.065) | 0.321 (0.065) |
| Β_3_ | (Standardised probe scale)^3^ | -0.233 (0.153) | -0.231 (0.153) |
| Β_4_ | Contingency | 0.08 (0.088) | 0.08 (0.089) |
| Β_5_ | Contingency.Standardised probe scale | -0.225 (0.101) | -0.219 (0.101) |
| Β_6_ | Contingency.(Standardised probe scale)^2^ | -0.223 (0.11) | -0.217 (0.11) |
| Β_7_ | Contingency.(Standardised probe scale)^3^ | -0.188 (0.213) | -0.192 (0.213) |
| Β_8_ | Treatment | -0.24 (0.079) | -0.272 (0.081) |
| Β_9_ | Measurement phase | 0.042 (0.028) | 0.044 (0.032) |
| Β_10_ | Treatment.Measurement phase | -0.055 (0.04) | -0.019 (0.045) |
| Β_11_ | Lever pressed | 0.113 (0.022) | 0.103 (0.028) |
| Β_12_ | Contingency.Lever pressed | -0.174 (0.032) | -0.175 (0.032) |
| Β_13_ | Lever pressed.Standardised probe scale | -1.261 (0.084) | -1.262 (0.084) |
| Β_14_ | Lever pressed.(Standardised probe scale)^2^ | 0.114 (0.099) | 0.115 (0.099) |
| Β_15_ | Lever pressed.(Standardised probe scale)^3^ | 1.134 (0.213) | 1.141 (0.213) |
| Β_16_ | Contingency.Lever pressed.Standardised probe scale | 0.67 (0.115) | 0.671 (0.115) |
| Β_17_ | Contingency.Lever pressed.(Standardised probe scale)^2^ | 0.237 (0.139) | 0.233 (0.139) |
| Β_18_ | Contingency.Lever pressed.(Standardised probe scale)^3^ | -0.41 (0.294) | -0.418 (0.294) |
| Β_19_ | Lever pressed.Treatment |  | 0.062 (0.029) |
| Β_20_ | Lever pressed.Measurement phase |  | -0.004 (0.028) |
| Β_21_ | Lever pressed.Treatment.Measurement phase |  | -0.07 (0.04) |
| **RANDOM PART** | |  |  |
| **Subject-level covariance matrix:** | |  |  |
| σ^2^*_v_*_0_ | | 0.028 (0.011) | 0.028 (0.011) |
| σ^2^*_v_*_01_ | | -0.003 (0.005) | -0.003 (0.005) |
| σ^2^*_v_*_1_ | | 0.009 (0.005) | 0.009 (0.005) |
| *σ^2^_v_*_02_ | | -0.008 (0.007) | -0.008 (0.007) |
| *σ^2^_v_*_12_ | | 0.003 (0.004) | 0.003 (0.004) |
| σ^2^*_v_*_2_ | | 0.006 (0.007) | 0.006 (0.007) |
| **Session-level covariance matrix:** | |  |  |
| σ^2^*_u_*_0_ | | 0.009 (0.002) | 0.008 (0.002) |
| σ^2^*_u_*_01_ | | -0.001 (0.002) | -0.001 (0.002) |
| σ^2^*_u_*_1_ | | 0.01 (0.003) | 0.011 (0.003) |
| *σ^2^_u_*_02_ | | -0.005 (0.004) | -0.005 (0.004) |
| *σ^2^_u_*_12_ | | 0.007 (0.004) | 0.007 (0.005) |
| σ^2^*_u_*_2_ | | 0.019 (0.012) | 0.019 (0.012) |
| **Trial-level variance:** | |  |  |
| σ^2^*_e_*_0_ | | 0.178 (0.003) | 0.178 (0.003) |

| **Response:** -1/√Latency(seconds) / **Link:** identity | | **Estimate (SE)** |
| --- | --- | --- |
| **No. of observations: n(subject)** = 16**; n(session)** = 96**; n(trial)** = 4600 | |  |
| **FIXED PART** | | **MODEL 8** |
| Β_0_ | Intercept | -1.36 (0.073) |
| Β_1_ | Standardised probe scale | -0.531 (0.092) |
| Β_2_ | (Standardised probe scale)2 | 1.048 (0.135) |
| Β_3_ | (Standardised probe scale)3 | 0.917 (0.197) |
| Β_4_ | Contingency | -0.011 (0.082) |
| Β_5_ | Contingency.Standardised probe scale | 0.411 (0.093) |
| Β_6_ | Contingency.(Standardised probe scale)2 | -0.375 (0.156) |
| Β_7_ | Contingency.(Standardised probe scale)3 | -0.726 (0.196) |
| Β_8_ | Treatment | -0.17 (0.086) |
| Β_9_ | Measurement phase | 0.04 (0.037) |
| Β_10_ | Treatment.Measurement phase | -0.075 (0.053) |
| Β_11_ | Measurement phase.Standardised probe scale | -0.084 (0.087) |
| Β_12_ | Measurement phase.(Standardised probe scale)2 | 0.049 (0.147) |
| Β_13_ | Measurement phase.(Standardised probe scale)3 | 0.268 (0.229) |
| Β_14_ | Treatment.Standardised probe scale | -0.233 (0.109) |
| Β_15_ | Treatment.(Standardised probe scale)2 | -0.004 (0.176) |
| Β_16_ | Treatment.(Standardised probe scale)3 | 0.275 (0.239) |
| Β_17_ | Measurement phase.Treatment.Standardised probe scale | 0.347 (0.123) |
| Β_18_ | Measurement phase.Treatment.(Standardised probe scale)2 | -0.304 (0.206) |
| Β_19_ | Measurement phase.Treatment.(Standardised probe scale)3 | -0.709 (0.322) |
| **RANDOM PART** | |  |
| **Subject-level covariance matrix:** | |  |
| σ^2^*_v_*_0_ | | 0.024 (0.009) |
| σ^2^*_v_*_01_ | | 0.006 (0.006) |
| σ^2^*_v_*_1_ | | 0.016 (0.008) |
| *σ^2^_v_*_02_ | | -0.002 (0.01) |
| *σ^2^_v_*_12_ | | 0.004 (0.009) |
| σ^2^*_v_*_2_ | | 0.035 (0.021) |
| **Session-level covariance matrix:** | |  |
| σ^2^*_u_*_0_ | | 0.01 (0.003) |
| σ^2^*_u_*_01_ | | 0.001 (0.003) |
| σ^2^*_u_*_1_ | | 0.003 (0.006) |
| *σ^2^_u_*_02_ | | -0.001 (0.005) |
| *σ^2^_u_*_12_ | | 0.001 (0.008) |
| σ^2^*_u_*_2_ | | 0.004 (0.019) |
| **Trial-level variance:** | |  |
| σ^2^*_e_*_0_ | | 0.167 (0.004) |

| **Response:** -1/√Latency(seconds) / **Link:** identity | | **Estimate (SE)** |
| --- | --- | --- |
| **No. of observations: n(subject)** = 16**; n(session)** = 96**; n(trial)** = 4026 | |  |
| **FIXED PART** | | **Model 9** |
| Β_0_ | Intercept | -1.383 (0.083) |
| Β_1_ | Standardised probe scale | 0.127 (0.093) |
| Β_2_ | (Standardised probe scale)^2^ | 0.416 (0.174) |
| Β_3_ | (Standardised probe scale)^3^ | 0.041 (0.224) |
| Β_4_ | Contingency | 0.123 (0.093) |
| Β_5_ | Contingency.Standardised probe scale | -0.141 (0.093) |
| Β_6_ | Contingency.(Standardised probe scale)^2^ | -0.096 (0.15) |
| Β_7_ | Contingency.(Standardised probe scale)^3^ | -0.225 (0.214) |
| Β_8_ | Treatment | -0.237 (0.097) |
| Β_9_ | Measurement phase | 0.055 (0.04) |
| Β_10_ | Treatment.Measurement phase | -0.033 (0.057) |
| Β_11_ | Measurement phase.Standardised probe scale | 0.177 (0.097) |
| Β_12_ | Measurement phase.(Standardised probe scale)^2^ | 0.214 (0.172) |
| Β_13_ | Measurement phase.(Standardised probe scale)^3^ | -0.691 (0.244) |
| Β_14_ | Treatment.Standardised probe scale | 0.091 (0.114) |
| Β_15_ | Treatment.(Standardised probe scale)^2^ | 0.085 (0.175) |
| Β_16_ | Treatment.(Standardised probe scale)^3^ | -0.186 (0.256) |
| Β_17_ | Measurement phase.Treatment.Standardised probe scale | -0.176 (0.137) |
| Β_18_ | Measurement phase.Treatment.(Standardised probe scale)^2^ | -0.245 (0.242) |
| Β_19_ | Measurement phase.Treatment.(Standardised probe scale)^3^ | 0.643 (0.348) |
| **RANDOM PART** | |  |
| **Subject-level covariance matrix:** | |  |
| σ^2^*_v_*_0_ | | 0.031 (0.012) |
| σ^2^*_v_*_01_ | | -0.005 (0.006) |
| σ^2^*_v_*_1_ | | 0.012 (0.006) |
| **Session-level covariance matrix:** | |  |
| σ^2^*_u_*_0_ | | 0.011 (0.002) |
| σ^2^*_u_*_01_ | | 0.001 (0.003) |
| σ^2^*_u_*_1_ | | 0.005 (0.005) |
| **Trial-level variance:** | |  |
| σ^2^*_e_*_0_ | | 0.177 (0.004) |

| **Response:** Lever pressed / **Link:** logit | | Estimate (SE) |
| --- | --- | --- |
| **No. of observations: n(subject)** = 16**; n(trial)** = 1024 | |  |
| **FIXED PART** |  | **Model 10** |
| Β_0_ | Intercept | -0.155 (0.144) |
| Β_1_ | Contingency | 1.09 (0.132) |
| Β_2_ | Treatment | 0.052 (0.187) |
| Β_3_ | Measurement phase | -0.441 (0.185) |
| Β_4_ | Treatment.Measurement phase | 0.441 (0.263) |
| **RANDOM PART** |  |  |
| σ^2^*_v_*_0_ | Subject-level variance | 0.001 (0.024) |

| **Response:** -1/√Latency(seconds) / **Link:** identity | | **Estimate (SE)** |
| --- | --- | --- |
| **No. of observations: n(subject)** = 16**; n(trial)** = 1024 | |  |
| **FIXED PART** | | **Model 11** |
| Β_0_ | Intercept | -1.339 (0.066) |
| Β_1_ | Contingency | 0.119 (0.078) |
| Β_2_ | Treatment | -0.205 (0.074) |
| Β_3_ | Measurement phase | -0.013 (0.042) |
| Β_4_ | Lever pressed | 0.186 (0.042) |
| Β_5_ | Contingency.Lever pressed | -0.293 (0.062) |
| Β_6_ | Treatment.Measurement phase | -0.115 (0.059) |
| **RANDOM PART** | |  |
| σ^2^*_u_*_0_ | Subject-level variance | 0.015 (0.007) |
| σ^2^*_e_*_0_ | Trial-level variance | 0.220 (0.010) |

| **Response:** -1/√Latency(seconds) / **Link:** identity | | **Estimate (SE)** |
| --- | --- | --- |
| **No. of observations: n(subject)** = 16**; n(trial)** = 1024 | |  |
| **FIXED PART** | | **Model 12** |
| Β_0_ | Intercept | -1.331 (0.073) |
| Β_1_ | Contingency | 0.116 (0.077) |
| Β_2_ | Treatment | -0.272 (0.09) |
| Β_3_ | Measurement phase | 0.021 (0.062) |
| Β_4_ | Lever pressed | 0.173 (0.067) |
| Β_5_ | Contingency.Lever pressed | -0.288 (0.062) |
| Β_6_ | Treatment.Measurement phase | -0.086 (0.09) |
| Β_7_ | Lever pressed.Treatment | 0.111 (0.086) |
| Β_8_ | Lever pressed.Measurement phase | -0.073 (0.084) |
| Β_9_ | Lever pressed.Treatment.Measurement phase | -0.032 (0.12) |
| **RANDOM PART** | |  |
| σ^2^*_u_*_0_ | Subject-level variance | 0.015 (0.006) |
| σ^2^*_e_*_0_ | Trial-level variance | 0.219 (0.010) |

| **Response:** Correct? / **Link:** logit | | **Estimate (SE)** |
| --- | --- | --- |
| **No. of observations: n(subject)** = 16**; n(session)** = 80**; n(trial)** = 12261 | |  |
| **FIXED PART** | | **Model 13** |
| Β_0_ | Intercept | 1.216 (0.178) |
| Β_1_ | Contingency | -0.46 (0.184) |
| Β_2_ | Treatment | -0.084 (0.212) |
| Β_3_ | Measurement phase | -0.175 (0.132) |
| Β_4_ | Treatment.Measurement phase | 0.202 (0.186) |
| Β_5_ | Reference stimuli | 0.697 (0.067) |
| Β_6_ | Contingency.Reference stimuli | 0.213 (0.091) |
| **RANDOM PART** | |  |
| σ^2^*_V_*_0_ | Subject-level variance | 0.097 (0.046) |
| σ^2^*_U_*_0_ | Session-level variance | 0.124 (0.029) |

| **Response:** Correct? / **Link:** logit | | **Estimate (SE)** |
| --- | --- | --- |
| **No. of observations: n(subject)** = 16**; n(session)** = 80**; n(trial)** = 12261 | |  |
| **FIXED PART** | | **Model 14** |
| Β_0_ | Intercept | 1.266 (0.181) |
| Β_1_ | Contingency | -0.461 (0.184) |
| Β_2_ | Treatment | -0.103 (0.22) |
| Β_3_ | Measurement phase | -0.212 (0.142) |
| Β_4_ | Treatment.Measurement phase | 0.144 (0.2) |
| Β_5_ | Reference stimuli | 0.571 (0.114) |
| Β_6_ | Contingency.Reference stimuli | 0.215 (0.091) |
| Β_7_ | Reference stimuli.Treatment | 0.049 (0.144) |
| Β_8_ | Reference stimuli.Measurement phase | 0.093 (0.131) |
| Β_9_ | Reference stimuli.Treatment.Measurement phase | 0.151 (0.185) |
| **RANDOM PART** | |  |
| σ^2^*_V_*_0_ | Subject-level variance | 0.096 (0.046) |
| σ^2^*_U_*_0_ | Session-level variance | 0.125 (0.029) |

| **Response:** -1/√Latency(seconds) / **Link:** identity | | **Estimate (SE)** |
| --- | --- | --- |
| **No. of observations: n(subject)** = 16**; n(session) = 80; n(trial)** = 12261 | |  |
| **FIXED PART** | | **Model 15** |
| Β_0_ | Intercept | -0.94 (0.07) |
| Β_1_ | Contingency | -0.12 (0.079) |
| Β_2_ | Treatment | -0.047 (0.081) |
| Β_3_ | Measurement phase | 0.135 (0.033) |
| Β_4_ | Reference stimuli | -0.241 (0.028) |
| Β_5_ | Contingency.Reference stimuli | 0.114 (0.038) |
| Β_6_ | Correct? | -0.421 (0.02) |
| Β_7_ | Reference stimuli.Correct? | 0.167 (0.031) |
| Β_8_ | Treatment.Measurement phase | -0.239 (0.047) |
| Β_9_ | Reference stimuli.Contingency.Correct? | -0.208 (0.043) |
| Β_10_ | Contingency.Correct? | 0.243 (0.027) |
| **RANDOM PART** | |  |
| σ^2^*_V_*_0_ | Subject-level variance | 0.021 (0.008) |
| σ^2^*_U_*_0_ | Session-level variance | 0.009 (0.002) |
| σ^2^*_e_*_0_ | Trial-level variance | 0.223 (0.003) |
